# Orthodontics and Temporomandibular Disorders: An Overview

**DOI:** 10.7759/cureus.47049

**Published:** 2023-10-15

**Authors:** Abdullah M Aldayel, Zainab J AlGahnem, Ibtehal S Alrashidi, Duaa Y Nunu, Afnan M Alzahrani, Wedad S Alburaidi, Fahad Alanazi, Abdulrahman S Alamari, Rami M Alotaibi

**Affiliations:** 1 Dental Clinic at King Khalid University Hospital, King Saud University, Riyadh, SAU; 2 Dentistry, Al-Ahsa Health Cluster, Al-Ahsa, SAU; 3 General Dentistry, Private Dental Clinic, Hail, SAU; 4 General Dentistry, Ministry of Health, Najran, SAU; 5 General Dentistry, Ministry of Health, Riyadh, SAU; 6 Dentistry, Qassim University, Buraydah, SAU; 7 General Dentistry, King Abdulaziz Medical City, Jeddah, SAU; 8 Family Dentistry, Ministry of Health, Riyadh, SAU; 9 General Dentistry, Ministry of Health, Jeddah, SAU

**Keywords:** temporomandibular joint, temporomandibular disorders, tmd, dental malocclusion, invisalign, orthodontic therapy, tmj disorders

## Abstract

The relationship between orthodontics and temporomandibular disorders (TMDs) constitutes a subject of paramount significance in dental and craniofacial health. This abstract embarks upon an in-depth examination of the intricate connection between orthodontic practices and TMD, primarily focusing on evaluating the impact of orthodontic treatment modalities on the health and functionality of the temporomandibular joint (TMJ).

This exploration elucidates the multifaceted interplay between orthodontic interventions and TMD by traversing a landscape of scholarly research and empirical investigations. The review draws from a broad spectrum of studies to analyze the potential influence of orthodontic treatments, which encompass occlusal adjustments and alterations in jaw positioning, on the development and management of TMD symptoms. The inquiry delves into the diverse range of TMD conditions, considering the implications of orthodontic techniques on occlusal stability, condylar alignment, and overall TMJ function.

Through a comprehensive synthesis of the available body of knowledge, this abstract aspires to equip dental practitioners, orthodontists, and researchers with a nuanced understanding of the complex dynamics that govern the relationship between orthodontics and TMD. This knowledge, in turn, offers a foundation for informed clinical decision-making and the formulation of effective treatment strategies for patients presenting with TMD symptoms. By shedding light on the intricate interactions between orthodontic procedures and TMJ health, this abstract contributes to the advancement of clinical practices, promoting improved patient outcomes and well-being in the context of both orthodontics and TMDs.

## Introduction and background

Occlusion has long been thought to be one of the main direct and/or indirect etiological factors producing temporomandibular disorders (TMDs) by clinicians and researchers [1]. TMD is a catch-all term for conditions involving masticatory muscle dysfunction and pain in conjunction with temporomandibular joints (TMJs) and adjacent tissues. It is a serious public health issue and the primary reason for nondental orofacial pain [2-4]. And behind chronic low back pain, it is the second most prevalent musculoskeletal ailment [5]. The anatomic connection between tooth position and jaw functions has long been recognized as the basis for the cause-and-effect relationship between occlusion and TMD, as has the fact that people with dental malocclusion had a higher frequency of TMD than people in the general population [6-7]. The development of TMJ pain and noises, headache, restricted mandibular opening, myofascial tenderness, and otological symptoms, collectively referred to as "Costen's syndrome," were all linked to occlusal changes and an increase in the overbite in 1934, according to otolaryngologist Dr. J. Costen [8]. Three decades later, Thompson [9] postulated that dental malocclusion might be to blame for the superior and posterior displacement of the condyle, arguing that TMD symptoms could be reduced by treating dental malocclusion. Studies have looked into occlusion and malocclusion-related factors as potential mechanisms for the emergence of TMD symptoms or signs [10]. For instance, a recent web-based study revealed that more than half of the currently available websites linked TMD to malocclusion or occlusal issues and advised treating these issues to treat TMD [11].

Numerous studies have examined the link between malocclusion and TMD, often with varying degrees of success. TMDs and malocclusions are two general names that cover a variety of various changes. While TMDs may include muscle and joint pain, disc displacement with or without joint noises, and pathologies that lead to an osseous remodeling of the TMJ, malocclusion, whether skeletal and/or dental, can be distinguished by disharmonies on the sagittal, transverse [12], and vertical planes [13].

In this review, we aim to provide an overview of the complex relationship between orthodontics and TMDs. By synthesizing the existing literature, we will explore the impact of malocclusion on TMD development, discuss treatment approaches, and highlight areas for further research. Through a comprehensive analysis, we seek to enhance our understanding of how orthodontics can contribute to the management and prevention of TMDs.

## Review

Research methodology

To conduct our research, we utilized PubMed, Google Scholar, Embase, and MEDLINE databases, employing the following keywords: "TMJ + malocclusion, orthodontic + TMJ, TMJ disorder, TMJ pain." We included all clinical trials and systematic reviews that matched our search criteria. Articles that were deemed inappropriate based on title alone were excluded. Additionally, a thorough evaluation of the abstracts helped us select the most relevant articles pertaining to our topic.

Transverse dental malocclusions

Unilateral posterior crossbite (UPCB) is the transverse malocclusion that is most frequently evaluated in terms of TMD, particularly in terms of TMJ clicking and myofascial discomfort. UPCB is more common in the younger population (5%-15%) and is thought to have a significant impact on the stomatognathic system [14,15]. In fact, it has been speculated that the aberrant occlusal contacts seen in patients with UPCB may have an impact on how the condyle and fossa are connected. Additionally, the asymmetry activation of the masticatory muscles caused by the different tooth contacts on the right and left sides may overload one side more than the other [16]. It has been hypothesized that individuals with UPCB may have a higher chance of developing TMJ clicking and myofascial pain as a result of these anatomical and functional alterations. However, there are also contradictory results in the literature [15]. Particularly, 10 years after the initial examination during adolescence, a relationship between UPCB and TMJ clicking was revealed, coupled with self-reports of TMJ clicking [17], although the original evaluation during adolescence found no association between the two conditions [14]. However, orthodontic treatment did not reduce the likelihood of subjects reporting TMJ sounds, raising doubts about the importance of occlusal factors and suggesting that anatomical factors, such as asymmetries in the glenoid fossa and condyle head, may have an impact on joint function in UPCB patients [18]. The results of a recent prospective study that examined the relationship between posterior crossbite, abnormal overjet, overbite, and TMJ clicking in a population of 903 people with a 30-year follow-up confirmed these conclusions and found no link between posterior crossbite and a higher risk of TMJ clicking [18]. It is interesting to note that while orthodontic treatment was not connected with self-reported TMJ clicking, emotional style and self-report of sleep bruxism were. It is clear that there is currently insufficient evidence to support the link between crossbite and TMJ clicking, given the fluctuating nature of the clicking, the significance of anatomical and psychological factors related to it, and the lack of effect on the clicking with correction of the crossbite. The relationship between UPCB and masticatory muscle discomfort has also been extensively studied, although the majority of these investigations neglected to take key factors such as muscular activity and psychological status into account [15]. A systematic electromyographic approach has been used to measure muscle activity in kids with and without UPCB to determine if the occlusal change causes asymmetrical muscle function or muscle overload [19]. According to the findings, children have asymmetric muscle activity that is unrelated to the presence of the UPCB [19]. Adult subjects with normal occlusion displayed symmetrical and balanced muscle activation, but those with significant malocclusions displayed asymmetrical muscular activation, according to studies [20,21]. These results imply that asymmetrical muscular activity is frequently observed in developing children without any signs or symptoms of TMD. However, in adults, asymmetric masticatory muscle activity may or may not be associated with the occurrence of persistent myalgia and muscular pain [22].

Sagittal dental malocclusions

There are few sporadic, weak, and inconsistent relationships with both sagittal and vertical malocclusions, according to a recent systematic review of the research on dental occlusion and TMD [23]. On the other hand, research has shown a connection between open bite, hyperdivergent development patterns, and degenerative TMJ diseases without proving a definite cause-and-effect relationship. Although there is still little evidence to support it, it is possible that the association between hyperdivergent growth patterns and TMJ disorders is caused by the latter conditions' early onset, which may result in abnormal condyle development [24,25].

A multifaceted etiology for TMD must be taken into account, and various factors, including comorbidities, oral parafunctions, psychosocial distress, muscle overload, somatic symptoms, and genetic markers, are supported by solid evidence as contributing factors [26]. Given that occlusal alterations may occasionally be a result of TMDs rather than their cause, the importance of occlusion in the genesis of TMD has not been thoroughly explored and should not be overestimated [27].

TMD and orthodontic treatment

It has also been asserted that orthodontic treatment may avoid or alleviate TMD as a result of the postulated connections between jaw malposition, occlusal variables, and TMD. The objective of treatment strategies for TMDs is to establish occlusal equilibrium by repositioning the mandibular condyles within the optimal location in the glenoid fossae or by achieving a presumed ideal occlusal or skeletal relationship. However, it is important to note that certain conventional orthodontic techniques, which disregard the principles of functional occlusion, have been identified as potential initiators and perpetuators of TMD symptoms and indications [28]. On the contrary, extensive scientific research conducted over the years consistently supports the notion that conventional orthodontic treatment has a neutral impact on the TMJ as well as TMDs in general. These studies provide robust evidence that traditional orthodontic interventions do not significantly contribute to the development or exacerbation of TMJ-related issues [29]. An example of this is seen in orthodontic/functional treatments targeting skeletal class II and class III malocclusions. Research has indicated that such treatments have not been linked to an increased risk of TMD during the treatment phase, nor a decreased risk of TMD following the completion of treatment [30-33].

Additionally, cone-beam computed tomography studies examining premolar extractions, commonly advised for maxillary teeth retractions, revealed indications of posterior condylar positioning following treatment. However, the clinical significance of these findings has been questioned as they did not demonstrate a higher prevalence of disc displacement [34]. The application of intermaxillary elastics in fixed orthodontic treatment has been the subject of recent finite element research. One study found that these elastic forces increased strain on the TMJ, particularly among class II patients [35]. However, the potential relationship between this increased strain and the development of TMD signs or symptoms remains unknown. In contemporary orthodontics, adult patients often opt for transparent aligner treatment due to its visual compatibility with their daily lifestyle [36]. More recent research involving clear aligners has indicated an increase in electromyographic activity of the masticatory muscles within six months of therapy [37,38]. Furthermore, thermoplastic orthodontic devices with complete occlusal coverage may be a favorable treatment option for individuals experiencing sleep bruxism, as they can safeguard tooth surfaces from dental wear [39]. However, one study conducted by Manfredini et al. [40] found no significant impact of invisible orthodontic retainers on the frequency of sleep bruxism in healthy individuals. Despite the absence of statistically significant differences between nights with and without the appliance, the authors noted a slight increase in masticatory muscle activity during sleep with the orthodontic retainer in place.

The effects of orthodontic treatment on TMDs have been investigated in several studies. One study found that after a month of regular orthodontic treatment, the proportion of patients reporting muscular soreness upon waking up increased. Examination of the TMJ and orofacial muscles revealed a substantial rise in the severity and number of painful areas. However, these symptoms were observed to be passing and gradually returned to normal over time [41]. In relation to clear aligners, recent studies have examined the impact of both passive and active aligners on the jaw. These studies have shown that while minor jaw soreness may occur for a brief period, none of the participants experienced overt TMD symptoms [42]. It is worth noting that the available evidence suggests that the presence of clear aligners does not diminish but rather increases masticatory muscle activity [43]. However, it is important to acknowledge that further studies with appropriate study design are needed to confirm these findings. Therefore, caution should be exercised when prescribing invisible orthodontic devices, such as clear aligners, to patients who may be at risk of experiencing jaw muscle soreness.

Additionally, it is important to recognize that orthodontic treatment cannot both induce and treat TMD, as indicated by the existing research [44]. Therefore, regardless of the kind of appliance used, there is no scientific basis for trying to prevent or treat TMD by achieving an "ideal" occlusion with orthodontic treatment [23].

Occlusion and orthodontics

Recent scientific developments have shifted the emphasis on the etiology of TMD away from a biomedical paradigm and toward a more intricate multifactorial biopsychosocial model that takes biological, psychological, and social aspects into account [44]. As a result, the definition of "occlusion" offers one potential explanation for the intense debates around the cause-and-effect link between occlusion and TMD [45]. The occlusion is generally seen from a mechanical perspective as either a static or dynamic connection between both the upper and lower dentition and the upper and lower jaw, which frequently deviates from what is thought of as "ideal." The occlusion, on the other hand, represents an excessively sophisticated specialized system of integrating neurological impulses emanating from periodontal, dental, and connective tissue receptors. The central nervous system (CNS) continuously develops this intricate informational structure to regulate and fine-tune jaw position or movements in response to the peripheral inputs [46]. As a result, the broad definition of occlusion encompasses both peripheral input (i.e., tooth-to-tooth contact) and the brain's interpretation of the same event. Therefore, how well a person adjusts to the occlusal and oral alteration that can follow from any dental treatment is largely dependent on the CNS changes (also known as "sensorimotor neuroplasticity"). Occlusal tactile acuity (OTA; the capacity to detect and identify small objects among the antagonist teeth throughout maximal intercuspation) plays a significant role in the process of occlusal adaptation/maladaptation because it is a highly variable trait across individuals and depends on a sophisticated information pathway [47]. The TMJ capsule, the muscles of mastication (muscle spindles), the dental pulp, and the mechanoreceptors in the periodontium are where the majority of the OTA's information is derived from [47]. The tactile input from the masticatory system during mastication offers sensory feedback that controls the occlusal force and triggers the reflex to open the jaw [47]. Increased OTA (i.e., heightened ability to sense tiny thickness among molars, compared to healthy controls) has been demonstrated to be related to reported wake-time dental parafunctions and TMD pain [48,49].

According to these findings, people who have parafunctional habits, as well as those who have TMD pain, may be more susceptible to occlusal alterations following dental treatments and, thus, more likely to acquire maladaptive behaviors. Contrarily, curiously, the OTA remained unchanged when myalgia was artificially induced in healthy participants devoid of TMD [50]. This may be attributed to the fact that the psychosocial domains, which are frequently compromised in TMD participants but not in healthy subjects with induced pain, have a substantial influence on the somatosensory function. Various people in various situations may interpret the same external signal (the thin space between antagonistic teeth) in different ways. This could be the result of a number of factors that affect exteroception and proprioception throughout the entire body. Perception constitutes a cognitive somatosensory encounter, and it relies not only on the strength of the stimulus but also on the mental state that controls how the stimulus is processed. Different sensory interpretation is primarily caused by top-down regulation of an input signal by higher-level brain centers and reconfiguration of the cortical regions [51]. The accurate understanding of the connection between "occlusion" and TMD thus seems essential, but the paradigm shift necessitates a transition to a more thorough meaning of the terms "occlusion" and "maxillo-mandibular relationship." The involvement of malocclusion in the pathophysiology of craniomandibular diseases should be minimized as there is no established causal link. Instead, clinicians should take patients' occlusal awareness and concerns seriously during general and dental examinations because a certain subset of patients may exhibit iatrogenic maladaptive behaviors.

## Conclusions

With the recently published findings, it is now necessary for dental professionals and orthodontists to abandon the conventional mechanical assessment of occlusion in favor of learning how to identify TMD and treat it with gentle, reversible treatments. When necessary, one should not be reluctant to enlist the assistance of other dental and medical specialists to provide multidisciplinary management. It is not unusual for patients to present having past experiences of orofacial pain in our routine clinical practice. Thus, a routine TMD-related assessment seems to be essential before beginning orthodontic therapy. Starting with a complete history, a thorough TMD examination to evaluate pain intensity, function hindrance from pain, and distress is required for orthodontists and general dentists. Clinicians and researchers can assess patients who may be susceptible to developing orofacial diseases using legitimate and trustworthy clinical and instrumental techniques for the diagnosis of disorders related to pain and dysfunction. The diagnostic criteria for TMD include a standardized diagnostic procedure. This tool includes two domains: Axis I for the physical assessment and Axis II for psychosocial evaluation. These will aid in gathering pertinent data to perform a standardized diagnosis and measure aspects of cognition, emotion, and behavior that may contribute to the aggravation and maintenance of pain and interfere with prognosis assessment and treatment planning. This gives general dentists and orthodontists the right resources to do differential diagnosis on patients with disorders that cause face discomfort, who typically are not good candidates to begin restorative or orthodontic therapy until the pain is controlled. It is typically advised to cease active treatment when TMD symptoms or signs emerge during orthodontic or dental therapy and to alleviate the discomfort as best as can. Once a patient is pain-free (or once the pain is successfully managed), dental and orthodontic therapy can be resumed as previously scheduled or, if necessary, changed based on the patient's condition.
